# An Analysis on the Detection of Biological Contaminants Aboard Aircraft

**DOI:** 10.1371/journal.pone.0014520

**Published:** 2011-01-17

**Authors:** Grace M. Hwang, Anthony A. DiCarlo, Gene C. Lin

**Affiliations:** 1 Department of the Office of Chief Engineer, The MITRE Corporation, McLean, Virginia, United States of America; 2 Department of Mechanical and Reliability Engineering, The MITRE Corporation, Bedford, Massachusetts, United States of America; 3 Department of West and International Redesign, The MITRE Corporation, McLean, Virginia, United States of America; George Mason University, United States of America

## Abstract

The spread of infectious disease via commercial airliner travel is a significant and realistic threat. To shed some light on the feasibility of detecting airborne pathogens, a sensor integration study has been conducted and computational investigations of contaminant transport in an aircraft cabin have been performed. Our study took into consideration sensor sensitivity as well as the time-to-answer, size, weight and the power of best available commercial off-the-shelf (COTS) devices. We conducted computational fluid dynamics simulations to investigate three types of scenarios: (1) nominal breathing (up to 20 breaths per minute) and coughing (20 times per hour); (2) nominal breathing and sneezing (4 times per hour); and (3) nominal breathing only. Each scenario was implemented with one or seven infectious passengers expelling air and sneezes or coughs at the stated frequencies. Scenario 2 was implemented with two additional cases in which one infectious passenger expelled 20 and 50 sneezes per hour, respectively. All computations were based on 90 minutes of sampling using specifications from a COTS aerosol collector and biosensor. Only biosensors that could provide an answer in under 20 minutes without any manual preparation steps were included. The principal finding was that the steady-state bacteria concentrations in aircraft would be high enough to be detected in the case where seven infectious passengers are exhaling under scenarios 1 and 2 and where one infectious passenger is actively exhaling in scenario 2. Breathing alone failed to generate sufficient bacterial particles for detection, and none of the scenarios generated sufficient viral particles for detection to be feasible. These results suggest that more sensitive sensors than the COTS devices currently available and/or sampling of individual passengers would be needed for the detection of bacteria and viruses in aircraft.

## Introduction

The potential for international airline passengers to transport infectious diseases into the United States is a serious concern. In 2003, the severe acute respiratory syndrome (SARS) virus was largely spread by air travelers and became a global epidemic; at least 18 countries on 5 continents were affected, resulting in over 8,000 cases and 774 fatalities [1]. One conservative estimate of the economic damage to Asian countries was calculated as $11 billion [2]. Influenza viruses that can be spread by air travelers have the potential to cause far greater harm [3], [4], [5]. Deliberate infection of passengers by terrorists is also a possible threat [6]. A potentially powerful tool to mitigate disease threats would be rapid and accurate detection of a variety of airborne infectious pathogens onboard commercial aircraft before passengers and crew deplane.

**Figure 1:**
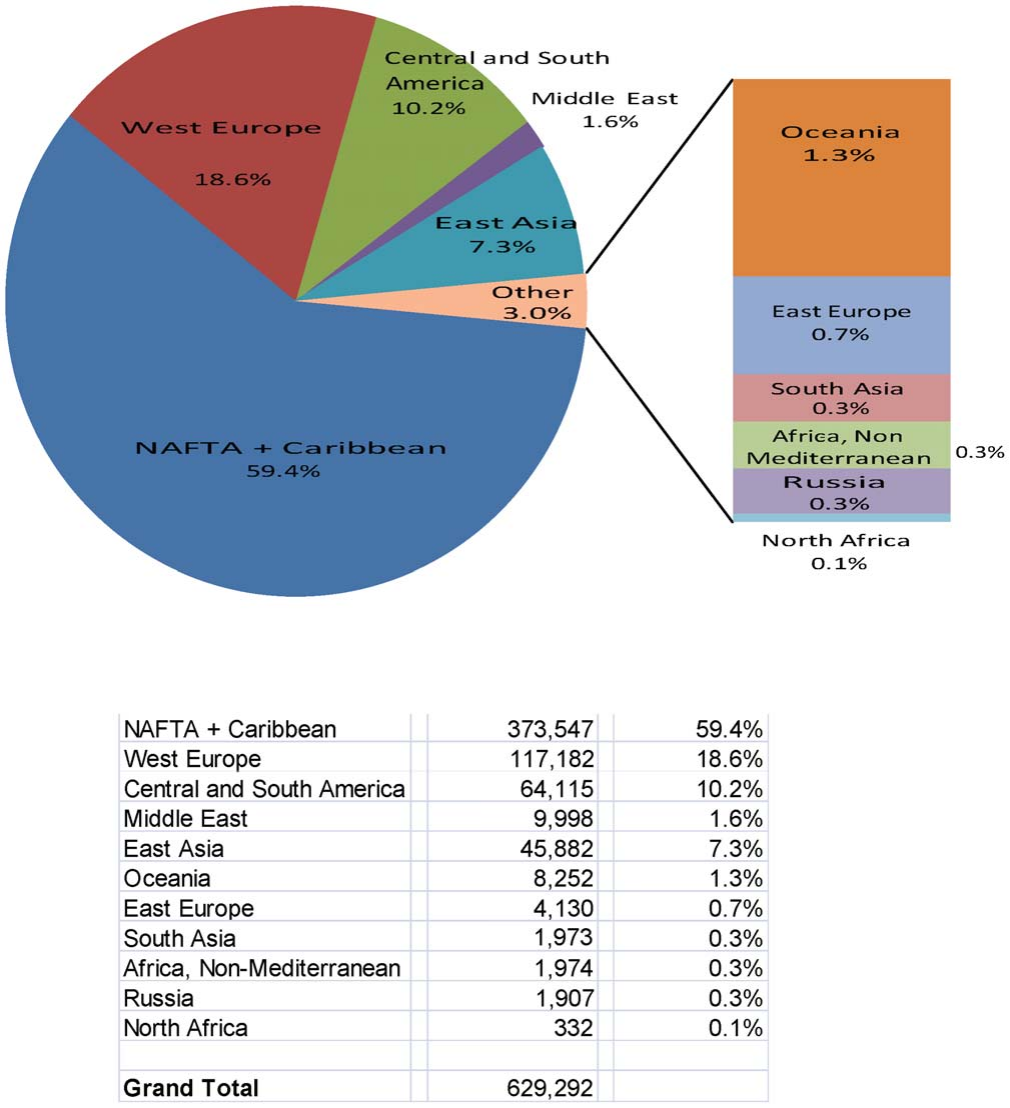
International Airline Arrivals into the United States, 2009. Source: Analysis of T-100 International Segment data, Bureau of Transportation Statistics, U.S. Department of Transportation.

We are interested in evaluating the feasibility of a rapid, reliable and miniature biosensor system that could be deployed onboard commercial aircraft. An appropriate biosensor would need to have a high probability of detection (P_D_>0.9) and a low probability of “false alarms” (i.e., P_FA_<10^−6^); to be capable of detecting airborne pathogens rapidly (in <3 hours for overseas international flights, in <1 hour for continental international flights) at non-lethal concentrations; to use minimal consumables so as to minimize system maintenance; to be relatively inexpensive to produce in large volumes; to be energy efficient, compact and lightweight (ideally each individual sensor would be cell phone-sized); and to be rugged enough to remain operable for at least twice the average working life of typical commercial aircraft. Based on these requirements, we apply a systems engineering approach by first establishing acceptable Type I (false positive) and Type II (false negative) error rates. To establish a Type I error rate, we consider that approximately 650,000 flights landed in the United States in 2009, suggesting that a sensor system with a P_FA_ of 10^−6^ would result in no more than one false alarm per year in the US (see Figure 1). Type II error rates should be based on the number of organisms commonly found in a commercial aircraft cabin today that would give rise to false negatives (e.g., contaminants in the air could cause one or more biosensing modalities to be impaired even in the presence of the pathogen) relative to the sensor's detection threshold. To establish a Type II error rate, we surveyed the literature and compiled microbial information from studies onboard aircraft (refer to Appendix of [3]). However, the granularity of the available data is insufficient to derive an acceptable Type II error rate.

**Figure 2:**
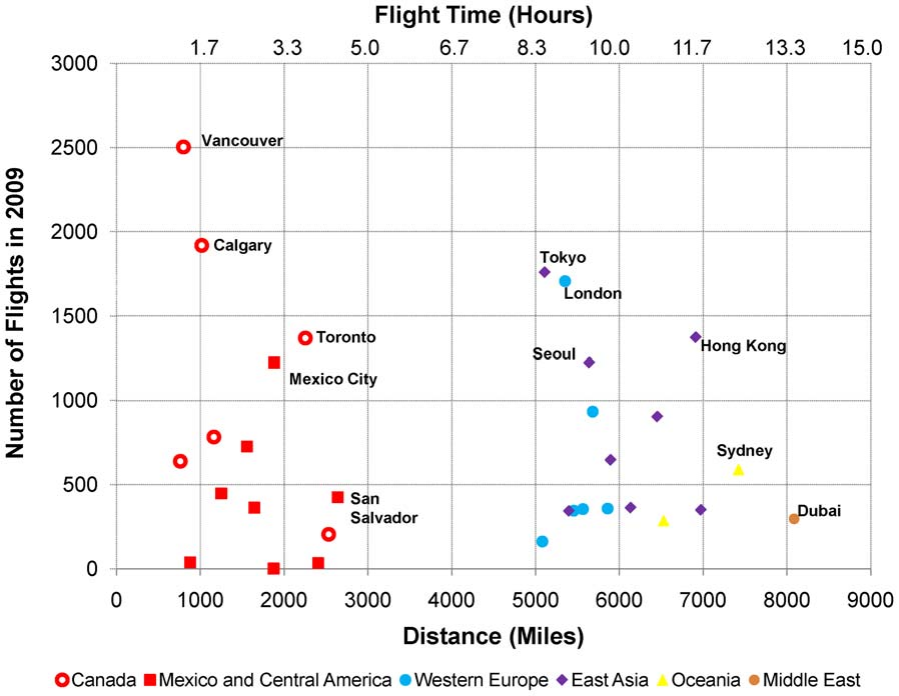
2009 Mean Flight Times to the San Francisco International Airport (SFO). Source: Analysis of T-100 International Segment data, Bureau of Transportation Statistics, U.S. Department of Transportation.

Our initial focus is to evaluate the feasibility of installing a biosensor system on overseas international flights (e.g., from China). We therefore analyzed the range of flight durations into a major U.S. international airport, the San Francisco International Airport (SFO) in 2009. As shown in Figure 2, flight durations from several international locations to SFO range from ∼1.5 to ∼13.5 hours. In particular, many flights from Canada to the United States with durations as short as 1.5 hours may have originated in China (i.e., Vancouver – ∼2504 flights per year; Toronto – ∼1368 flights per year). We therefore designed model scenarios around a minimum aerosol sampling duration of 1.5 hours.

To understand contaminant transport and airflow patterns inside an aircraft cabin, prior researchers have used computational fluid dynamics (CFD) models to predict airflows with [7] and without [8], [9] simulated passengers. Some of these studies were based on measurements using 3D ultrasonic anemometers [10] and particle image velocimetry systems [11]. Measurements of airflow were conducted using mock airliner cabins with [10], [12], [13], [14], [15], [16], [17] and without [18] simulated passengers (e.g., heated manikins or heaters). Chemical contaminants such as SF_6_ tracer gas and bio-simulant particles such as mono-dispersed (0.7 micron) di-ethyl-hexyl-sebacat [DEHS] have been modeled using the Reynolds averaged Navier-Stokes equations based on the Renormalized Group (RNG) κ–ε turbulence model [12], [17], [19]. In particular, to determine optimal sensor placement in a mock wide-body aircraft cabin section, CFD simulations have been used to model different chemical release conditions (continuous or discrete doses) and release rates (10^−7^, 10^−6^, or 10^−5^ m^3^/s [12], [19]). These studies reported that the optimal sensor placement in a wide-body airliner cabin would be in the middle of the ceiling for simulated sensor sensitivity ranging from 0.01 parts per million (ppm) to 10 ppm [12], [19]. Although these previous studies are insightful and valuable, they do not directly address whether sufficient human exhalations are released into the aircraft cabin for onboard biosensors to detect the presence of harmful particles. Earlier work also failed to correlate tracer gas concentration with the number of viral particles per cubic meter of cabin air or infectivity.

In the present study, we consider exhaled particles from regular breathing, coughing and sneezing. Since infectious airborne particles are typically under 20 microns [4], here we restrict our simulation and analysis to expellants with diameters under 20 microns. To establish a medically relevant cough scenario, we surveyed studies that measured cough frequencies from patients with respiratory conditions for up to 24 continuous hours. These results are summarized in Table S1 for asthma [20], [21], chronic obstructive pulmonary disease [22], [23], [24], chronic cough [25], [26], [27], [28], cystic fybrosis [29], idiopathic pulmonary fibrosis [30], primary ciliary dyskinesia [31], pulmonary tuberculosis [23], and pneumonia [23]. Based on these results, we simulate 20 coughs per hour as a representative cough frequency in infected passengers. To establish a plausible sneeze scenario, we assume that a typical infectious passenger can sneeze from 4 to 50 times per hour, where the highest sneeze frequency approaches that of a “super spreader” — a term that can refer to persons with “high values of cough and/or sneeze frequency, elevated pathogen concentration in respiratory fluid, and/or increased respirable aerosol volume per expiratory event such that their pathogen emission rate is much higher than average” [32]. In addition to particles expelled during coughing and sneezing, particles exhaled during regular breathing were also accounted in each scenario. To establish a plausible breathing scenario, the distribution and concentration of particles exhaled during breathing were taken from studies that employed aerodynamic particle sizers [33], combined with data previously reported in the literature (see Methods for details). Collectively, we simulated three types of scenarios: (1) breathing and coughing, (2) breathing and sneezing, and (3) inhaling only through the nose and exhaling only from the mouth.

## Results

**Figure 3:**
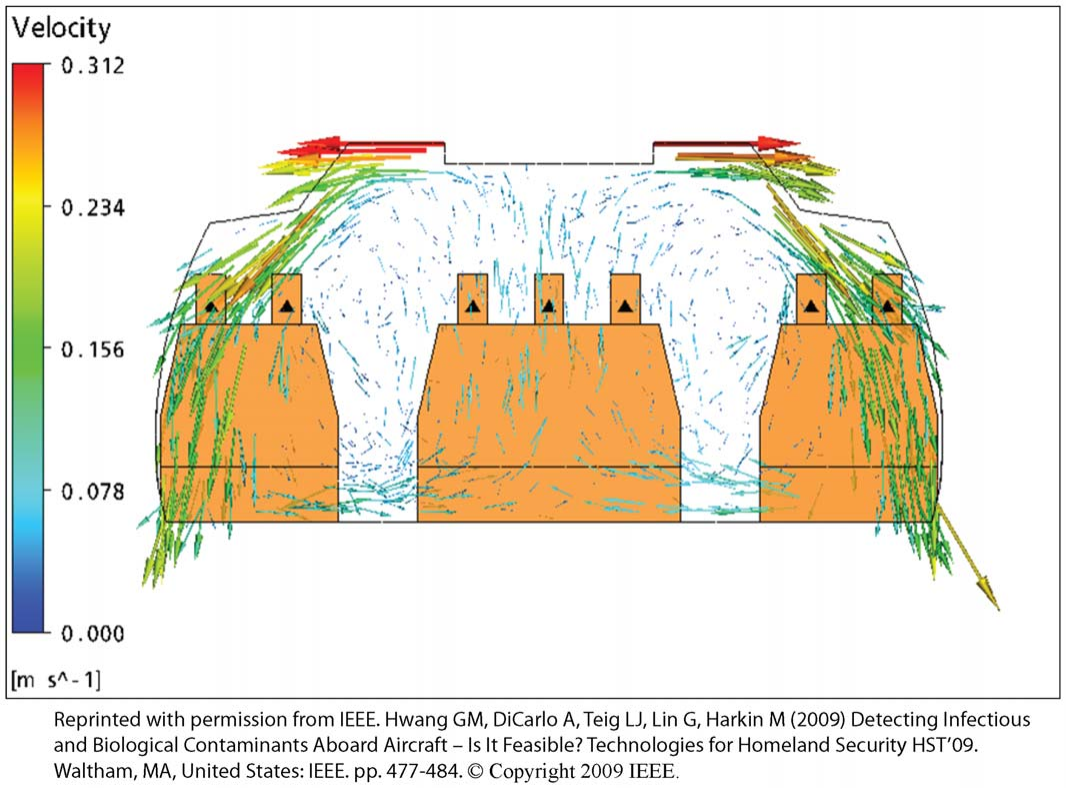
4” Mesh Velocity Contour.

To verify that the temperature and velocity gradients computed by our CFD model are consistent with previous work by Chen *et al.* [17], we compared the velocity profiles from a 4” mesh and found good agreement. As shown in Figure 3, the airflow occurs primarily transverse to the main axis of the cabin. This is in part due to the absence of simulated passenger traffic in the aisle. Furthermore, the CFD aircraft cabin flow fields presented here are consistent with results presented by independent investigators [17] and the U.S. Federal Aviation Administration (FAA) [34].

**Figure 4:**
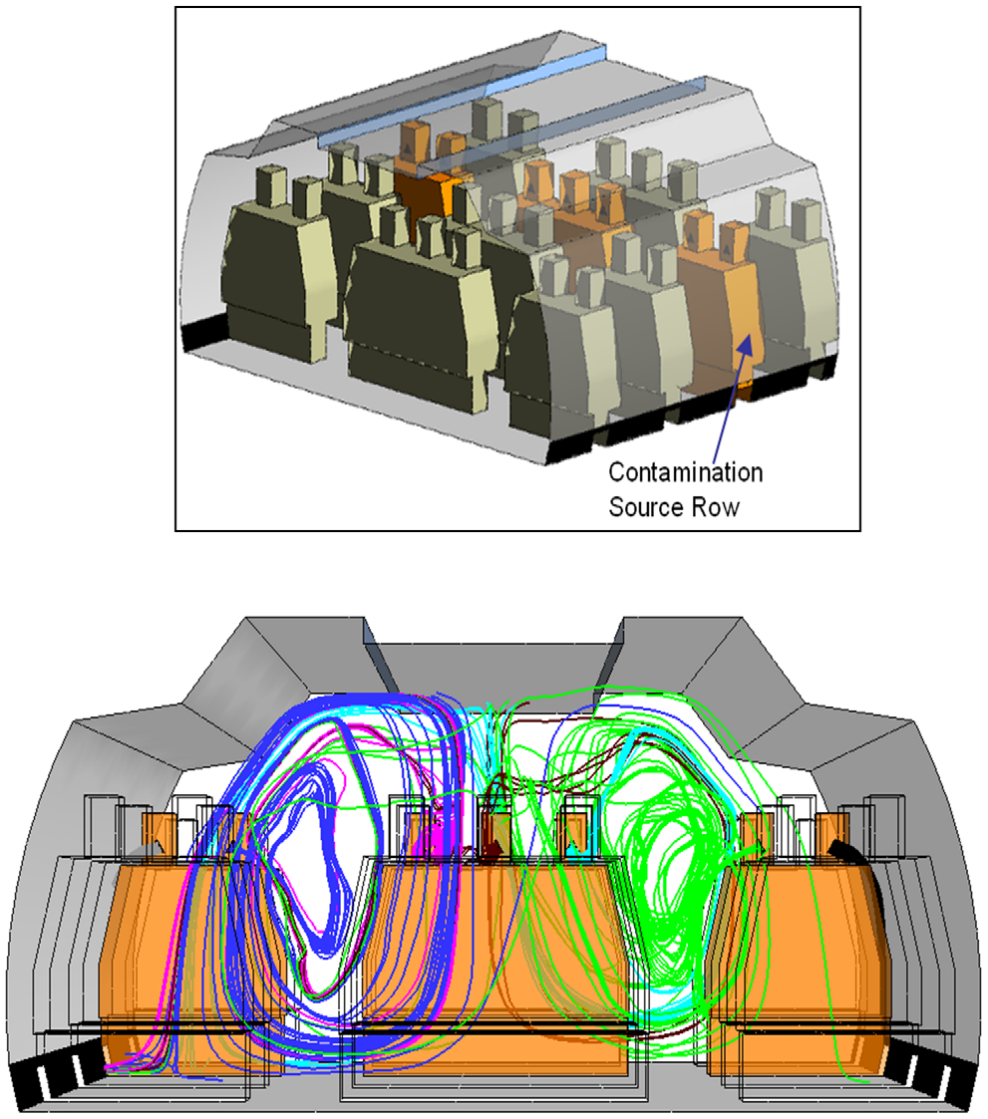
Infected Passenger Schematic. Seat Position from Left to Right: LK FED BA. Contaminant Streamlines Shown for Scenario 1 Breathing and Coughing.

In our CFD simulations, we specified some number of infected passengers whom we considered to be contaminant producers (Figure 4, second-to-last row, shown in orange). The locations of the mouths of these passengers are the initial positions of simulated exhaled particles. To visualize the transverse extent of exhalant trajectories during a breathing-and-coughing scenario, we show streamlines originating at these positions that represent the flow of particles across the cabin (Figure 4, bottom). Each of the infected passengers released the same amount of contaminant within each case simulated.

To assess the feasibility of onboard bio-detection equipment for each tested scenario, the particles exhaled from each infected passenger were simulated under steady-state conditions (equilibrium, well-mixed) and the airborne particles concentrations were computed based on their diameter size using specifications from COTS aerosol collectors. For purposes of classification, we refer to particles that are between 1 and 20 microns in diameter as “bacterial” and particles less than 1 micron as “viral”. We then compared the accumulated biomass to the limit of detection (LoD) of COTS biosensors. Case A was simulated with seven infected passengers, each breathing 20 times a minute across all scenarios, where scenario 1 simulates breathing and coughing, scenario 2 simulates breathing and sneezing, and scenario 3 simulates breathing only. Cough and sneeze rates were set to 20 and 4 exhalation events per hour, respectively. The steady-state masses in case A were computed to be 3.43×10^−9^ kg, 5.12×10^−9^ kg, and 3.07×10^−9^ kg for scenarios 1, 2, and 3, respectively (refer to Table 1, Case A, row a). These masses correspond to approximately 6×10^6^ bacterial and 6×10^6^ viral particles in scenario 1, over 1.0×10^7^ bacterial and over 8×10^3^ viral particles in scenario 2, and over 1.0×10^5^ bacterial and over 1.7×10^5^ viral particles in scenario 3 (see Table 1, Case A, row b).

**Table 1:** Estimates of collectable biological particles.

Scenarios:	(1) Breathing & Coughing		(2) Breathing & Sneezing		(3) Breathing Only	
	Bacterial	Viral	Bacterial	Viral	Bacterial	Viral
**Case A. Seven Super spreader**						
a) Accumulated mass at steady state (kg)	3.43×10^−9^		5.12×10^−9^		3.07×10^−9^	
b) Total particles at steady state	6.469×10^6^	6.570×10^6^	1.082×10^7^	8,589	1.088×10^5^	1.782×10^5^
c) Biological particles per m^3^	113.02	14.41	189	<1	1.9	0.39
d) No. of collectable biological particles per m^3^	102.66	5.14	172	<1	1.7	0.14
e) No. of collectable viable particles in 90 minutes at 0.3 m^3^/min	2,771.79	138.69	4,635	<1	46.62	3.76
f) No. of collectable particles in 90 minutes at 0.3 m^3^/min	5.897×10^6^	2.351×10^6^	9.862×10^6^	3,073	9.919×10^4^	6.375×10^4^
**Case B. One Super spreader**						
a) Accumulated mass at steady state (kg)	7.97×10^−10^		1.15×10^−9^		6.98×10^−10^	
b) Total particles at steady state	1.502×10^6^	1.525×10^6^	2.425×10^6^	1,925	2.479×10^4^	4.059×10^4^
c) Biological particles per m^3^	26.24	3.35	42	<1	0.43	0.09
d) No. of collectable biological particles per m^3^	23.84	1.19	38	<1	0.39	0.03
e) No. of collectable viable particles in 90 minutes at 0.3 m^3^/min	643.55	32.20	1039	<1	10.62	0.86
f) No. of collectable particles in 90 minutes at 0.3 m^3^/min	1.369×10^6^	5.458×10^5^	2.210×10^6^	689	2.260×10^4^	1.452×10^4^
**Case C. One Super spreader**						
a) Accumulated mass at steady state (kg)			3.45×10^−9^			
b) Total particles at steady state			1.525×10^6^	1,210		
c) Biological particles per m^3^			27	<1		
d) No. of collectable biological particles per m^3^			24	<1		
e) No. of collectable viable particles in 90 minutes at 0.3 m^3^/min			653	<1		
f) No. of collectable particles in 90 minutes at 0.3 m^3^/min			1.390×10^6^	433		
**Case D. One Super spreader**						
a) Accumulated mass at steady state (kg)			8.52×10^−9^			
b) Total particles at steady state			1.571×10^6^	1,247		
c) Biological particles per m^3^			27	<1		
d) No. of collectable biological particles per m^3^			24	<1		
e) No. of collectable viable particles in 90 minutes at 0.3 m^3^/min			673	<1		
f) No. of collectable particles in 90 minutes at 0.3 m^3^/min			1.432×10^6^	446		

To obtain the biological particles per cubic meter of cabin space, we applied two factors. First, the total number of particles at steady-state (row b) was divided by the total volume of air in the four-row aircraft cabin section, which was 26.9 m^3^. Second, the bacterial particles were multiplied by a viable fraction of 4.7×10^−4^ while viral particles were multiplied by an estimated viable fraction of 5.9×10^−5^ to obtain biological particle counts per cubic meter, respectively (see Table 1, Case A, row c) [35]. To estimate the number of collectable viable particles as a function of particle size (i.e., bacterial versus viral), the values in row c were multiplied by aerosol collector efficiencies which account for the fraction of particles collected with respect to the total number of particles present. Specifically, based on values from a commercially available collector (OMNI 3000, Kansas City, MO), we applied collector efficiencies of 0.91 and 0.357 for the bacterial and viral particles, respectively [36]. Finally, the total cumulative numbers of collectable viable biological particles in a 90-minute continuous sampling interval were calculated based on a typical collector flow rate (i.e., 0.3 m^3^ per minute) (see Table 1, Case A, row e). Because the viability coefficients germane to estimating bacterial and viral particle counts were based on limited estimates from a single study [35], we included the total number of collectible particles in Table 1, Case A, row f as an upper bound on detection. The values in Table 1, Case A, row f were not used in this analysis because it is highly unlikely that a person would exhale 100% infectious bacteria or virus even in states of high bacterial or viral shedding.

To determine the feasibility of using onboard biosensors to detect the presence of airborne particles, the numbers of collectable viable particles in 90 minutes (Table 1, Case A, row e) from all cases were compared with the LoD in mature, commercially available biosensors [37]. Commercial biosensors that rely on nucleic acid-based polymerase-chain reaction (PCR) amplification are known to have limits of detection of less than 10 copies, and generally provide a time-to-answer in 60 minutes. However, because manual sample preparation is outside the scope of this analysis and antibody-based biosensor systems can generally provide a time-to-answer in under 20 minutes, we only considered non-PCR biosensor systems here. The typical LoD for a COTS antibody-based biosensor system ranges from 10^3^–10^4^ organisms per test (manufacturers typically refer to bacterial organisms as colony-forming units and viral organisms as plaque forming units) [37]. Therefore, based on these analyses, COTS collectors and biosensors would be sufficiently sensitive to detect bacterial targets in a 90-minute sampling interval for only scenarios 1 (breathing and coughing) and 2 (breathing and sneezing). Scenario 3 (breathing only) generated fewer than 10^3^ bacterial particles. Further, none of the scenarios generated sufficient viral particles for detection to be feasible. Note, however, that there are significant limitations and unknowns involved in quantifying viral particles without direct data gathered from field tests (see Discussion).

To examine the sensitivity of our analysis, we decreased the number of infectious passengers from seven to one in case B for scenarios 1 though 3. All other input parameters were identical to case A. The numbers of collectable viable particles in 90 minutes (Table 1, Case B, row e) from all cases were compared with 10^3^ organisms per test. In contrast to case A, only scenario 2 (breathing and sneezing) generated sufficient numbers of bacterial particles (i.e., more than 10^3^) for onboard detection to be feasible. The remainder of case B simulation results were well beneath the 10^3^ organisms per test, suggesting that onboard detection would not be feasible.

To test the robustness of our analysis, we expanded on scenario 2. Case C was simulated based on one infected person breathing up to 20 times a minute and sneezing 20 times per hour, while case D was simulated based on one super spreader breathing up to 20 times a minute and sneezing 50 times per hour (see Methods for details). In both cases C and D, we found that over 600 bacterial particles would be detected in a 90- minute sampling interval (Table 1, Case C and D, row e), while no more than one viral particle would be detected. Taken together, these estimates suggest that onboard detection would not be feasible for bacterial- or viral- particles in cases C and D.

To confirm that equilibrium conditions are indeed reached early during a flight, we calculated the time to reach steady state. Note, however, that transient characteristics are primarily governed by airflow. The following concentration rates are provided for completeness, not to imply that the equilibrium time depends upon the rate of contaminant introduction. First, we used known data to calculate the approximate expellant volume of particles under 20 microns for one cough (∼2.04×10^−7^ml), one sneeze (∼5.27×10^−5^ml), and one breath (1.24×10^−8^ml). (See Methods for details.) We also modeled seven passengers who either cough 20 times per hour in scenario 1, sneeze 4 times per hour in scenario 2, or simply breathe 20 times per hour in scenario 3. This allowed us to calculate total per-passenger particle-generation rates (in kg per second) of 4.67×10^−12^, 7.04×10^−12^, and 4.15×10^−12^, respectively (Table 2). Taking the ratio of the steady state concentrations for scenarios 1 through 3 (Table 1, Case A [kg per cabin volume]: 3.43×10^−9^, 5.12×10^−9^, 3.07×10^−9^) to the per-passenger particle-generation rate, we arrived at approximately 12 minutes to reach steady state for scenarios 1 through 3. Twelve minutes is well within a typical flight time and under the 90-minute sampling time interval.

**Table 2:** Expiratory parameters per passenger.

Scenarios (case)	Expiratory Description	Concentration (kg/m^3^) †	Average Expellants (kg/s) §	Average Volume Exhaled (L/s)
1 (A&B)	Breathing and Coughing –<20 breaths per minute and 20 coughs per hour	2.51×10^−8^	4.67×10^−12^	0.186
2 (A&B)	Breathing and Sneezing – <20 breaths per minute and 4 sneezes per hour	4.13×10^−8^	7.04×10^−12^	0.171
3 (A&B)	Breathing Only –20 breaths per minute	2.49×10^−8^	4.15×10^−12^	0.167
2 (C)	Breathing and Sneezing –<20 breaths per minute and 20 sneezes per hour	1.07×10^−7^	1.99×10^−11^	0.186
2 (D)	Breathing and Sneezing –<20 breaths per minute and 50 sneezes per hour	2.30×10^−7^	4.94×10^−11^	0.215

Notes:

§ Expellant Density Assumed to be Same as Water (998 kg/m^3^).

† Air Density Represents a Pressurized Cabin at an Altitude of 7000 Feet (0.81 kg/m^3^) [50].

**Figure 5:**
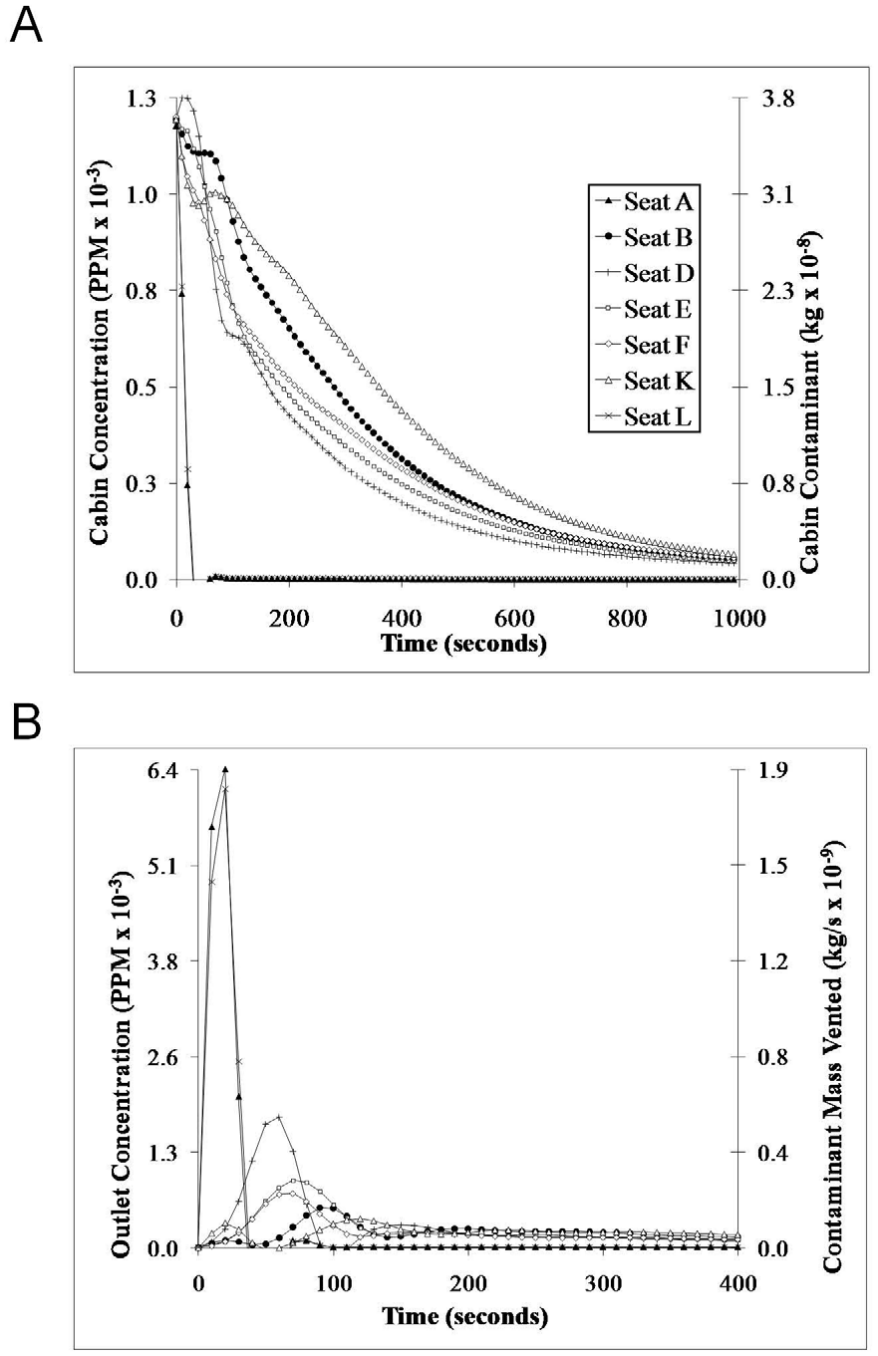
Time History of Contaminant Transport. (a) Contaminant Concentrations in the Airliner Cabin. (b) Contaminant Mass Flow Rate at the Outlet Vents.

Due to the temporal characteristics associated with sneezing and coughing, a time history study is required to understand the movements of contaminant particles released during these expiratory events (Figure 5). In the transient case presented here, each passenger sneezes once and the fluid expelled at each seat location was tracked with regard to the time to transport the contaminant to the outlet vents. The resultant history of the contaminant in the cabin is computed in terms of contaminant mass (kg) and concentration in ppm relative to the air (Figure 5A). The contaminant concentration shown in Figure 5A represents the contaminant mass divided by the cabin volume. As a result, these values are not the measure of local values of contamination at each seat position in the cabin, some of which would show higher contaminant concentrations. Rather, the numbers represent average concentrations across each four-row cabin section illustrated in Figure 4.

The contaminant mass flow rate at the outlet vent is shown in Figure 5B. We observe that contaminants emitted from window seat positions (Figure 4, seats L and A) enter the outlet ventilation much faster than those emitted from other seats. It is possible that the flow from the window seats may be too transient for a ceiling-mounted collector above the central seats to aggregate enough particles to achieve reliable identification from those passenger seated in L or A. To mitigate this relatively low sampling rate, the collector may require a high inflow rate. Further, this suggests that a collector's effectiveness may increase when the collector is placed at the outlet vent near the window seat position. This may not be the most appropriate sensor location for collecting non-window-seat emissions, because the contaminant concentration becomes substantially diluted by the time it reaches the outlet, as shown by the second y-axis in Figure 5B.

Overall, the findings for each of these three scenarios were similar and consistent with the findings of Chen's laboratory [12], [17]. The general air flow characteristics are two big vortices caused by the ventilation system that generates a recirculation swirl on each side of the aircraft. Specifically, particle emissions from the two window seat passengers (Figure 4, seats L and A) were the least circulated contaminants within the cabin (see Figure 5A) and entered the outlet vents quickly (see Figure 5B). In contrast, particles emitted by the passengers seated at the center or aisle seats lingered in the cabin and were not transported as effectively to the outlet vent compared to particles that originated from the window seats (see Figure 5B). The particles emitted from the aisle seat passengers near the windows (seats B and K) tend to linger longer because it enters the recirculation zone at a location where the air is pushing the particles away from the vents. The particles emitted from the aisle passengers in the center seats (seats D and F) enter the recirculation zone at a location where it is driven towards the vents. These results should not be applied broadly to all wide-body aircraft because the simulations were based on a representative Boeing 767 aircraft cabin.

## Discussion

Three scenarios of infectious “super spreader” passengers were investigated, consisting of states of extreme coughing, extreme sneezing, and regular breathing. Our principal finding was that the steady-state bacteria concentrations in aircraft would be high enough to be detected in the case where seven infectious passengers are exhaling under scenarios 1 (breathing and coughing) and 2 (breathing and sneezing), and where one infectious passenger is actively exhaling in scenario 2. Breathing alone failed to generate sufficient bacterial particles for detection, and none of the scenarios generated sufficient viral particles for viral detection to be feasible.

This is consistent with a recent study by Fabian *et al*. who found that only 33% of infected persons exhale detectable viral RNA. Fabian et al. sampled directly from a breathing apparatus with Teflon filters and identified the collected particles by RNA extraction [38]. Unlike this direct sampling method, the aircraft cabin is designed for rapid dilution by turning over the air volume approximately 20 times per hour. Taken together, these findings provide further support for the view that it is difficult to collect and detect viral particles directly from cabin air using autonomous collector and biosensor systems.

It is important to realize that infectious particles can be emitted during regular breathing. Particles released during regular breathing are predominantly under 1 micron in diameter [38], small enough to enter the human alveolar region and upper respiratory tract. Some subjects can exhale more contaminant particles from breathing than from coughing: consider that the average person breathes 20 times a minute and each breath may contain 0.5 liters of air. Although one cough may release up to 3.56 liters of air, the typical cough frequency of a sick person is usually less than 50 times per hour. The rate of exhalation for breathing is therefore significantly higher for breathing than for coughing. In this study, we assumed that exhaling passengers are not covering their mouths during coughing and sneezing; however, airline passengers are likely to cough or sneeze into their hands or elbows, though most do not cover their mouths when breathing. This differential in mitigating behaviors by passengers enhances the possibility of asymptomatic airborne transmission. Interestingly, observational studies conducted on the novel H1N1 virus reported a wide range of asymptomatic infection rates: from <10% among households in Germany and Canada [39], [40] to over 90% in India [41]. For influenza virus alone, multiple modes of transmission have been hypothesized [42], [43], and there is limited information on viral shedding from asymptomatic persons. However, we believe that airway transmission via regular breathing cannot be ruled out as a putative transmission pathway for communicable respiratory diseases. Further investigation of this pathway is especially critical given recent data on the number of particles exhaled during normal breathing [44].

Other studies suggest different values for the amount of bacteria and viruses produced by sneezing and breathing. A related study on *staphylococcus aureus* dispersion (11 subjects) from sneezes reported 2.83 and 3.24 colony-forming units (CFU) per cubic meter per minute of *S. aureus* and coagulase-negative staphylococci, respectively [45]. If we assume that 6 CFU represent 6 particles in the 2–4 micron range based on data from the Duguid sneeze study [35], and that one sneeze approximates 3.56 liters of air in one second, then this would suggest that the viable fraction of infectious bacteria per sneeze is 8.1×10^-6^. This is less than the viable fractions used to compute the total number of collectable viable particles in Table 1, row e. Another study of influenza (13 symptomatic subjects) reported that one person generated up to 20 influenza particles per minute during breathing [38]. The same study reported 87% of exhaled particles were <1 micron with particle counts ranging from 61–3,848 and 5–2,756 per liter of air for particle sizes ranging from 0.3–0.5 and 0.5–1 microns, respectively. So in one minute, if a person breathed at a rate of 10 liters per minute, a possible range of 660 to 66,040 particles under 1 micron could be generated, giving rise to influenza-containing particle fractions ranging from 0.03 to 0.0003. The Fabian study did not address the viability of influenza particles; rather, the analyses were based on particle identification using nucleic acid amplification. Thus, it is not possible to deduce a viability coefficient from the results of their study. Note that the viable fraction in our study is 5-fold lower than the influenza particle fractions reported by Fabian *et al.* Given these large discrepancies and the overall scarcity of relevant data in the literature, it would be beneficial to conduct experiments using non-infectious virus (and/or 120 nm polystyrene beads that exhibit size characteristics comparable to influenza viruses) to empirically test the number of collectible influenza particles emitted in a 90-minute interval within a mock aircraft section.

One may notice that the viral particle counts from the breathing-and-sneezing scenario are quite low relative to those computed for the breathing-and-coughing and breathing-only scenarios. This discrepancy is due to the data source employed in our calculation. Specifically, all sneeze data was based on a single 1946 study by Duguid [35], which did not observe any particles of <1 micron diameter. In addition, there were fewer breaths per minute in the breathing-and-sneezing scenario compared to the breathing-only scenario. It would be beneficial to repeat the sneeze-related analysis using newer data from sneeze experiments that used sick persons as subjects.

The study has additional limitations, including the assumption that there is no longitudinal mixing more than four rows from the infectious passenger along the length of the airliner cabin. Indeed, recent experimental evidence suggests that biological particles can be detected more than four rows away from the emission source [46]. This far-field transmission study was conducted in a wide-body mock aircraft cabin section under ambient pressure and representative flight ventilation conditions. The biological particles were emitted from a handheld mister and may not be entirely representative of human exhalation. A further limitation of the present analysis is that detection does not imply infection. Viral particles are known to degrade quickly (from minutes to hours) outside host environments [43], bacteria and spores can have longer survival times (>five weeks) [47]. Infection is additionally confounded by the host's immune system; the findings in the present study do not address infectivity. Collectively, the limitations imposed by the parameters used in this study were intended to create the most optimistic case for a biosensor system that could collect and detect viable bacterial and viral pathogens. Equally important, but completely omitted in the analyses presented here, is the P_FA_, which must be minimized in any deployed biosensor system. Given the low steady-state particle concentrations of viral pathogens in cabin air, even in an optimistic case where seven passengers in a row are actively exhaling particles, future studies should consider the feasibility of rapidly detecting infectious particles directly from human exhalation using hand-portable devices and well-targeted sampling schemes (e.g., direct sampling of sputum or nasopharyngeal fluid).

## Materials and Methods

### The Model

The models developed in this work closely follow the work conducted by Chen *et al.* [12], [16], [17], [48]. The difference in the present work is that the source producing the representative-sized contaminants more closely resembles a passenger breathing and coughing, breathing and sneezing, or simply regular breathing. ANSYS CFX commercial software was utilized for the CFD simulations. The software computes the contaminant transport via advection and diffusion, as shown in equation 1.
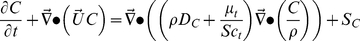
(1)where


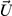
  =  velocity (m/s)

C  =  concentration, mass of contaminant per unit volume of air (kg/m^3^)

ρ  =  mixture density, mass per unit volume (kg/m^3^)

S_C_  =  volumetric source term, contaminant per unit volume of air per unit time (kg/m^3^ s)

D_C_  =  kinematic diffusivity (m^2^/s)

µ_t_  =  turbulent viscosity (Pa s)

Sc_t_  =  turbulent Schmidt number (non-dimensional)

Equation 1 assumes that particles follow airflow streamlines. As a result, the particles' velocity is not computed. This method of modeling is valid when the particle diameter is relatively small and particle dispersion is not important [49]. The majority of airborne particles small enough to enter the human respiratory tracts are less than 20 µm in diameter [42] and are also small enough to remain suspended in the airflow. According to the Stokes number, it is reasonable to state that particles less than 75 microns will stay suspended and follow the lazy particle model [49]. By assuming that no particle settles onto a surface and all particles stay suspended and possibly continue to the collector is not entirely realistic but it is sufficient to prove this paper's point; that particle detection with the commercially available biosensors surveyed is not practical.

The CFD model we employed is representative of the airflow in a Boeing 767 airliner cabin. A renormalized group kinetic energy-dissipation (RNG κ-ε) turbulence model, assuming air as an ideal gas and reference pressure of 1 atmosphere, was used because of the model's reasonable accuracy and low computational cost [16]. The boundary conditions used in these simulations are listed in Table 3. At the inlet, a mass flow rate of 0.313 kg/s was applied. This was chosen on the basis of information that the plane's environmental control system (ECS) provides 20 cubic feet per minute (cfm) of air per passenger [50]. We needed to apply a boundary condition to the outlet; applying a standard “outlet” condition resulted in slower runtime and program warnings due to the methods the program uses to apply an outlet condition. It proved more effective to apply an “opening” condition with a negative pressure to simulate the air being drawn from the cabin into the outlet vents and recirculation system. The model was tested using each of the 8”, 4”, and 2” meshes according to the settings described in Table 3. Several mesh sizes were considered, with the 4” mesh providing the greatest accuracy in a reasonable amount of computation time. Due to turbulence and minor variations in airflow it was difficult to track precise values for single points or nodes, but viewing the overall velocity profile for each mesh showed good agreement from trial to trial [3].

**Table 3:** Aircraft cabin boundary conditions and exhaled air for each scenario.

Total Volume Modeled = 26.9 m^3^	Temperature (°C)	Velocity and Flow Characteristics
Supply Air Velocity	19.3	0.312 m/s
Cabin Wall	24	0 m/s
Passenger Surface	31	0 m/s
Exhaled Air per Passenger for Scenario 1 – Breathing and Coughing	35	1.86×10^−4^ m^3^/s
Exhaled Air per Passenger for Scenario 2 – Breathing and Sneezing	35	1.71×10^−4^ m^3^/s
Exhaled Air per Passenger for Scenario 3 – Breathing Only	35	1.67×10^−4^ m^3^/s

### Scenario Descriptions

Three types of scenarios were simulated: (1) breathing and coughing, (2) breathing and sneezing, and (3) simply inhaling through the nose and exhaling from the mouth. Scenario 1 employed a combination of breathing and coughing where the simulated infected passenger coughed 20 times per hour and breathed the remainder of the time at a rate of 20 breaths per minute. Scenario 2, like scenario 1, employed a combination of breathing and sneezing where the simulated infected passenger sneezed four times per hour and again breathed at a rate of 20 times per minute. In scenario 3, the simulated infected passenger simply breathed 20 times per minute. The amount of air volume exhaled per expiratory event was set to 0.5 liters per breath, and 3.56 liters per cough or sneeze. The simulated duration of expiratory events was 3 seconds for each breath, and 1 second for each cough or sneeze. The particle concentrations, shown in Table 2, reach steady-state conditions in approximately 12 minutes, which is well within the 90-minute sampling duration relevant to this study. Two cases were explored within each scenario described above: an extreme case in which all seven passengers seated in a row were sick and a case in which only one seated passenger was sick. Two additional cases (C and D) were explored for scenario 2 (sneezing and breathing) in which one seated passenger sneezed 20 and 50 times per hour, respectively. Using these input conditions, the average amount of fluid (from saliva) and average air expelled per person during scenarios 1 through 3 are also summarized in Table 3 in columns 4 and 5, respectively.

### Particle Generation Estimates

**Figure 6:**
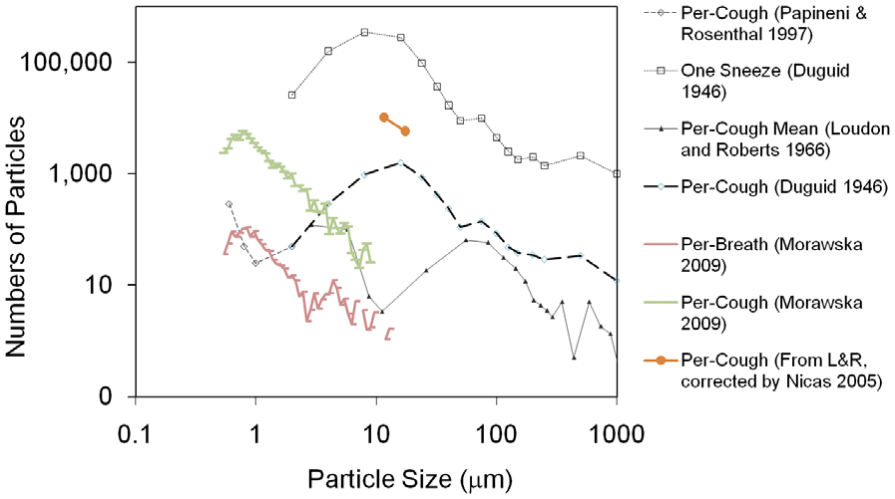
Particle Size Distribution from Human Exhalations.

To estimate the number of particles emitted by mouth from human exhalations as a function of particle size, we graphically summarized a few prior studies that measured particle size distributions (Figure 6): Morawska [33] used a custom-designed, pre-filtered, carefully humidity-controlled wind tunnel and reported mean particle concentrations based on aerodynamic particle sizer counts for up to 50 particle diameter ranges between 0.542 and 20 microns (number of subjects [N] = 15). Six different scenarios were tested, including breathing through the nose (b-n-n), breathing through the nose and exhaling from the mouth (b-n-m), whispered counting, voiced counting, and coughing. Figure 6 depicts b-n-m and cough, as they were the only two scenarios relevant to the current work. Loudon and Roberts [51] used an air-tight box and Millipore filter air sampler and reported per-cough mean particle counts to be 120, 100, 6.2 and 1.7 for diameter ranges (µm) 2–5.8, 5.8–11.6, 11.6–17.4, 17.4–20, respectively (N = 3). Papineni and Rosenthal [52] used a combination of electron microscopy and an optical particle counter inside a biological safety cabinet and reported per-cough mean particle counts to be 290, 50, 25, 35, 10, 10 for diameter ranges (µm) <0.6, 0.6–0.8, 0.8–1.0, 1.0–1.5, 1.5–2.0, and 2.0–2.5, respectively (N = 5). We concatenated the Loudon and Roberts and Papineni and Rosenthal particle size distributions and inferred a total particle count of 647.9 per one cough, of which 410 particles had diameters smaller than 2 µm and 237.9 particles had diameters between 2 and 20 µm (Figure 6). We then compared the concatenated particle size distribution with the more recent cough distribution and elected to use Morawska's data for particles under 10 microns and Loudon and Roberts' data for particles between 11.6 and 20 µm. Therefore, the cough analyses presented in this work are based on data presented by Morawska [33], and Loudon and Roberts as corrected by Nicas for evaporative losses [32], [51].

To arrive at a reasonable total liquid volume exhaled per cough from particles under 20 microns in diameter, we estimated the total volume per exhalation by applying equations described by Nicas [32] using particle size distribution from two of Morawska's experimentation scenarios: (i) breathing normally through the nose and exhaling through the mouth (b-n-m) and (ii) coughing [33]. Briefly, *V*_20_ refers to total number of particles in each range of diameters up to 20 microns, multiplied by the mean particle volume in each respective range [32], as shown in equation 2.
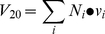
(2)where *N*_i_ represents the number of particles observed in the *i* ^th^ diameter range without assuming any evaporative losses, and 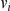 is the mean volume of a particle in the corresponding *i* ^th^ diameter range. Because we employed data supplied by Morawska, it was necessary to normalize mean concentration data into units that apply to one exhalation event. To calculate the number of particles from one breath, 0.5 liters of air was multiplied to the mean particle concentration, which assumed complete release of tidal volume from a typical person [53]. To calculate the number of particles from one cough, 3.56 liters of air was multiplied by the mean particle concentration; the 3.56 liter value was based on the forced expiratory volume in one second reported from normal subjects [20].

In this work, we calculated the mean volume of particles in each corresponding diameter range, based on the minimal (d_min_) and maximal (d_max_) diameters within each range, consistent with prior work. These calculations assume a uniform distribution of particle diameters within each size range.
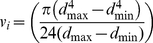
(3)


Unlike prior work, our study did not assume 50% evaporative loss for diameter ranges for cases where inputs came from the Morawska dataset. In diameter ranges where Morawska did not report any particles due to insufficient instrument signal (i.e., >10 microns for the cough scenario), we employed older data from Loudon and Rosenthal (see two points on Figure 6).

The particular aerodynamic particle sizer employed by the Morawska study (TSI model 3312A) had counting efficiencies that deviated from 100%; only a fraction of all the particles that passed through the sizer were counted. The sizer's counting efficiency was (%) 30, 100, and 60, respectively, for particle sizes (µm) 0.5, 0.9, and 5 [54]. Because up to 50 possible diameter ranges were used to estimate *V*_20_, we elected not to correct for particle counting inefficiencies as a function of diameter ranges due to the sparseness of available correction factors.

Based on the methods described in equations 2 and 3, we estimated a *V*_20_ of 1.247×10^-8^ ml for one breath, and a *V*_20_ of 2.04×10^-7^ ml for one cough. Prior calculations by Nicas [32], using data from Loudon and Rosenthal, estimated a *V*_20_ of 6×10^-8^ ml for one cough. We note that if we had not combined the recent Morawska data with the older Loudon and Rosenthal data, the *V*_20_ for a cough would be 1.88×10^-7^ ml using only the Morawska cough data. Since the intent of this work was to compute the most optimistic case for biosensor detection, this work was based on concatenated data, which resulted in a higher volume of expellants per cough event.

To estimate the number of particles exhaled in one sneeze, we considered Duguid (1946), who used a combination of food dye, oiled slides, micrometry and three different test chambers to measure the average number of droplets and droplet-nuclei generated when subjects sneezed (∼10^6^ particles), coughed (∼5×10^3^ particles), and spoke loudly (∼250 particles). As shown in Figure 6, Duguid reported per-sneeze mean particle counts to be 26×10^3^, 160×10^3^, 350×10^3^, 280×10^3^, and 97×10^3^ for diameter ranges (µm) 1–2, 2–4, 4–8, 8–16, and 16–24, respectively. In addition, Duguid reported per-cough mean particle counts to be 50, 290, 970, 1.6×10^3^, 870 for diameter ranges (µm) 1–2, 2–4, 4–8, 8–16, and 16–24, respectively (refer to Figure 6). Since the present study is most interested in particles under 20 microns, we computed the ratio of total sneezed particles under 20 microns to total coughed particles under 20 microns by assuming that the 16–24 microns diameter range is uniformly distributed across size and therefore only counted 50 percent of the particles within 16–24 microns to arrive at particle counts within 16–20 micron. Using this logic, we found that a sneeze is a factor of 258 times larger than a cough in terms of particle counts (i.e., ratio of 864,500/3,345). Therefore, to calculate the volume of expellants from one sneeze, we simply multiplied the mass of one cough at every diameter range under 20 microns by 258, which resulted in 5.27×10^−5^ml, while the volume of air released was set to 3.56 liters. To estimate the mean particle count under 20 microns diameter in a sneeze for the CFD simulation, we assumed that one sneeze corresponded to approximately 8.645×10^5^ particles, and that the particle size distribution followed those reported by Duguid [35], also shown in Figure 6.

In summary, the amounts of fluid expelled during a single expiratory event based on *V*_20_ for breathing, coughing, or sneezing were 1.247×10^−8^ml, 2.04×10^−7^ml, and 5.27×10^−5^ml, respectively.

### Particle Collection Estimates

To compute the total number of particles at steady-state for each scenario modeled, the steady-state masses were divided by the weighted sum of the unit masses expelled by the respective expiratory activities. For the breathing and coughing scenario, the steady-state mass was divided by the weighted sum of one cough and one breath based on 12 minutes of sampling, where 12 minutes was the amount of time for the model to reach a steady-state. Specifically, a passenger coughing 20 times per hour would on average emit 4 coughs per 12 minutes (20 coughs/hr ×1 hr/60 min ×12 min). The same passenger breathing 20 times per minute would emit ∼96 breaths in the same 12 minutes. The weighted sum of the particle distribution contributions from breathing and coughing were ∼96% and ∼4%, respectively. Therefore, the *V*_20_ (described by equations 2 and 3) for one breath was converted to mass and multiplied by 96%, while the *V*_20_ for one cough was converted to mass and multiplied by 4%. Similarly, for the breathing and sneezing scenario, the steady-state mass was divided by the weighted sum of one sneeze and one breath based on 12 minutes of sampling. For example, a passenger sneezing 4 times per hour would emit on average 0.8 sneezes in 12 minutes. The same passenger breathing 20 times per minute would emit just under 98 breaths in 12 minutes. In this model, the weighted contributions from breathing and sneezing were ∼99.2% and ∼0.8%, respectively. Thus, the *V*_20_ for one breath was converted to mass and multiplied by 99.2%, while the *V*_20_ for one sneeze was converted to mass and multiplied by 0.8%. For the breathing-only scenario, the steady-state mass was simply divided by the mass of a breath as converted from *V*_20_.

To estimate the number of collectable biological particles, collection efficiencies (CE) were gathered from independent wind-tunnel testing conducted by the U.S. government using a range of polystyrene latex beads between 0.5 and 8 microns in diameter [36]. The aerosol collection rate employed by Kesavan *et al.* (2006) was 277 liters per minute, which is sufficiently close to 300 liters per minute. We applied CE from 3 and 0.5 microns beads corresponding to 0.91 and 0.357 for the bacterial and viral calculations, respectively.

To estimate the number of viable bacterial and viral particles, we surveyed the literature and found very few studies that quantified viability as a function of particles exhaled by size. We therefore relied on a very old Duguid (1946) study that measured the number of organisms per ml of saliva as a function of particle size. Duguid reported that particle diameters ranging from 1–2 to 2–4 microns corresponded to viability coefficients of 5.9×10^−5^ and 4.7×10^−4^, respectively. For simplicity, the viability coefficient for particles with diameters ranging from 1–2 microns was applied to viral particles, while the viability coefficient for particles with diameters ranging from 2–4 microns was applied to bacterial particles.

## Supporting Information

Table S1 Compilation of cough statistics. Notes: Chronic cough can include asthma, gastrooesophageal reflux, eosinophilic bronchitis, chronic obstructive pulmonary disease and chronic bronchitis. § Italics represent cases where median values are reported by primary literature. † Study reports cough frequency in cough seconds per hour. In this table, 1 cough second is assumed to be the equivalent of one cough. NR denotes not reported.
(0.59 MB TIF)
