# Supplementary figures and images for: An Analysis on the Detection of Biological Contaminants Aboard Aircraft
Source: PLoS One. 2011 Jan 17;6(1):e14520. doi: 10.1371/journal.pone.0014520 (PMC3022008; doi:10.1371/journal.pone.0014520)

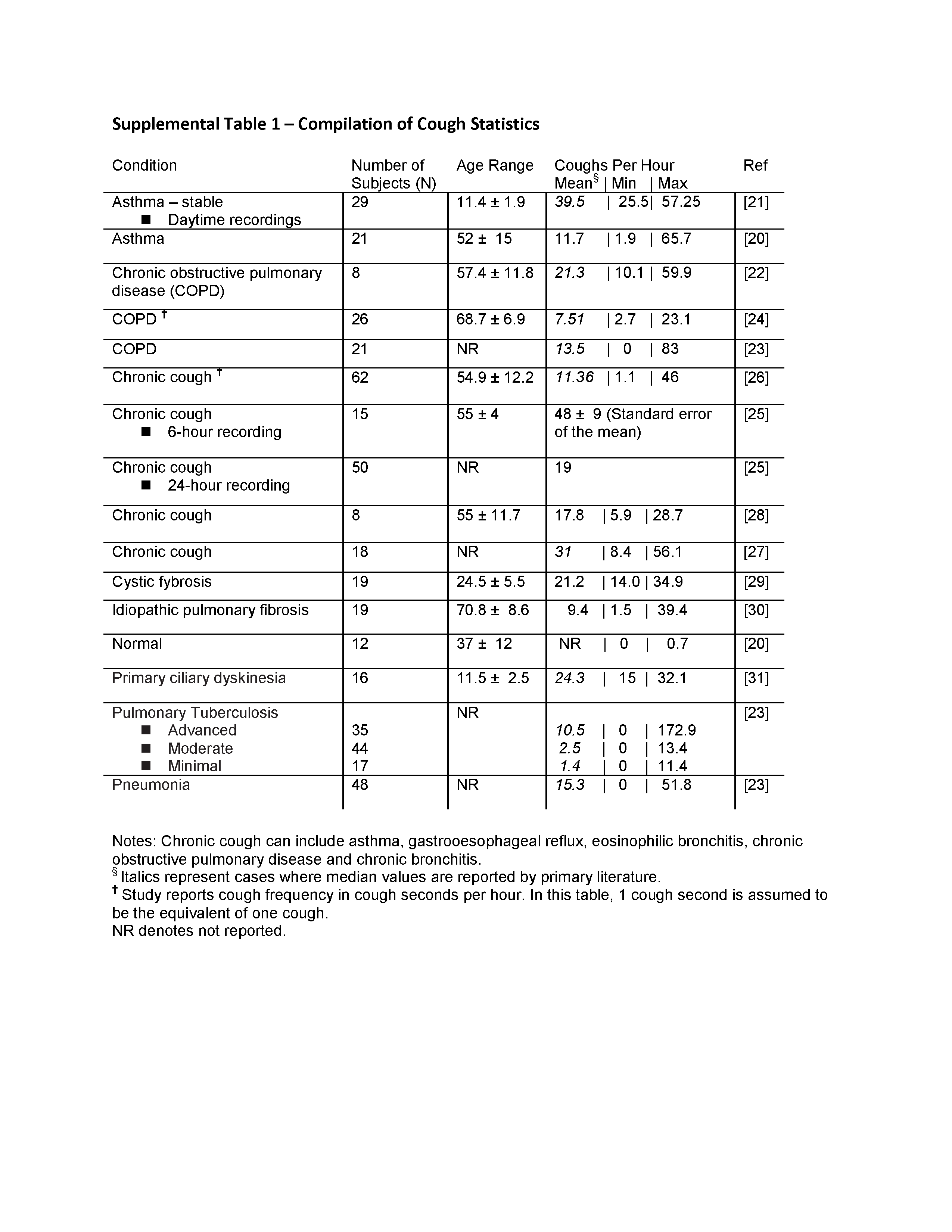

Supplement: Table S1 — Compilation of cough statistics. Notes: Chronic cough can include asthma, gastrooesophageal reflux, eosinophilic bronchitis, chronic obstructive pulmonary disease and chronic bronchitis. § Italics represent cases where median values are reported by primary literature. † Study reports cough frequency in cough seconds per hour. In this table, 1 cough second is assumed to be the equivalent of one cough. NR denotes not reported. (0.59 MB TIF) [file pone.0014520.s001.tif]
